# Turning conceptual systems maps into dynamic simulation models: An Australian case study for diabetes in pregnancy

**DOI:** 10.1371/journal.pone.0218875

**Published:** 2019-06-27

**Authors:** Louise Freebairn, Jo-An Atkinson, Nathaniel D. Osgood, Paul M. Kelly, Geoff McDonnell, Lucie Rychetnik

**Affiliations:** 1 ACT Health, Canberra, Australia; 2 The Australian Prevention Partnership Centre, Sax Institute, Sydney, Australia; 3 University of Notre Dame, Sydney, Australia; 4 Decision Analytics, Sax Institute, Sydney, Australia; 5 Sydney Medical School, University of Sydney, Sydney, Australia; 6 Computer Science, University of Saskatchewan, Saskatoon, Canada; 7 Department of Community Health … Epidemiology, University of Saskatchewan, Saskatoon, Canada; 8 Medical School, The Australian National University, Canberra, Australia; Yale University Yale School of Public Health, UNITED STATES

## Abstract

**Background:**

System science approaches are increasingly used to explore complex public health problems. Quantitative methods, such as participatory dynamic simulation modelling, can mobilise knowledge to inform health policy decisions. However, the analytic and practical steps required to turn collaboratively developed, qualitative system maps into rigorous and policy-relevant quantified dynamic simulation models are not well described. This paper reports on the processes, interactions and decisions that occurred at the interface between modellers and end-user participants in an applied health sector case study focusing on diabetes in pregnancy.

**Methods:**

An analysis was conducted using qualitative data from a participatory dynamic simulation modelling case study in an Australian health policy setting. Recordings of participatory model development workshops and subsequent meetings were analysed and triangulated with field notes and other written records of discussions and decisions. Case study vignettes were collated to illustrate the deliberations and decisions made throughout the model development process.

**Results:**

The key analytic objectives and decision-making processes included: defining the model scope; analysing and refining the model structure to maximise local relevance and utility; reviewing and incorporating evidence to inform model parameters and assumptions; focusing the model on priority policy questions; communicating results and applying the models to policy processes. These stages did not occur sequentially; the model development was cyclical and iterative with decisions being re-visited and refined throughout the process. Storytelling was an effective strategy to both communicate and resolve concerns about the model logic and structure, and to communicate the outputs of the model to a broader audience.

**Conclusion:**

The in-depth analysis reported here examined the application of participatory modelling methods to move beyond qualitative conceptual mapping to the development of a rigorously quantified and policy relevant, complex dynamic simulation model. The analytic objectives and decision-making themes identified provide guidance for interpreting, understanding and reporting future participatory modelling projects and methods.

## Introduction

This paper contributes to the current knowledge gap about the development from qualitative to quantitative modelling [1]. It examines the detailed implementation of the analytic processes and practical strategies used to convert the qualitative systems maps into a rigorous and policy relevant dynamic simulation model. Dynamic simulation models are quantified, computer-based representations of complex systems that draw on best available evidence and provide a decision support tool to conduct policy experiments and forecast potential impacts. The models enable working hypotheses of causal pathways to be explicitly and quantitatively operationalised to evaluate the effectiveness of potential interventions, or combinations of interventions, via computer simulation before they are implemented in the real world [2–6].

This paper provides a qualitative analysis of the stakeholder deliberations and decisions that occurred within an Australian health sector participatory modelling case-study. This case study applied the participatory approach to the development of a multi-method (or hybrid; these terms are explained below) dynamic simulation model focusing on diabetes in pregnancy. We present the findings together with real-world examples of some of the core questions and decisions made, to inform health service researchers, policy makers and modellers who may be considering undertaking participatory modelling projects. The findings detail important aspects of project implementation, and the types of input from end-user participants. This includes the feedback, critiques, issues raised, and questions asked by stakeholders as part of their engagement in the participatory modelling process; their analytical and material contributions to model development and peer-to-peer learning; and their role in the process of identifying and selecting different forms and sources of evidence. We also report on the intellectual and practical challenges experienced by the core model building team—and strategies used to overcome them, as well as the overall challenges and significant opportunities arising from the participatory process itself.

### Background

Knowledge created through application of the scientific method requires effort to translate into action [7]. Knowledge mobilisation is defined as a dynamic and iterative process that includes synthesis, dissemination, exchange and application of knowledge to improve health, provide more effective health services and products and strengthen the health care system [8]. It is widely acknowledged that using research evidence for policy and practice is an emergent and context dependent process, that relies on relationships, and can be time consuming and lack clear policy direction particularly in the face of complexity that characterises many of our persistent health and social problems [9–13].

The many synergies of combining evidence-informed policy principles with systems science methods are increasingly recognised [14]. Systems science encompasses a range of approaches that can be used to explore and understand public health problems as complex systems; in order to intervene more effectively and adapt to each particular context [15–19]. Key elements of a systems science approach include synthesising diverse knowledge and evidence, exploring the potential for non-linear relationships between contributing factors, and identification of unanticipated emergent behaviour of the complex systems (including policy resistance) [15, 19–22].

The collaborative exploration of a complex issue or problem using systems thinking can generate a conceptual system map which reflects the qualitative, group understanding of the complex issue [23, 24]. These qualitative maps and models can engender a high degree of ownership and consensus about the nature of the problem, as they are based on the collective expertise of the participants involved [25]. However, the practical application of these maps in exploring and testing hypotheses about the impact of policy intervention options is limited [25, 26]. Such hypothesis testing and comparison of the impacts of alternative scenarios relies on subsequent rigorous quantification of the components, connections and relationships that comprise the system using methods such as dynamic simulation modelling [25–27]. Simulation modelling allows experiments to be conducted to see how a system behaves under different conditions and scenarios [22, 28]. The postulated theory of causation is refined and shaped through the participatory process of model building [6]. The process can enable health policy and practice decisions makers to sharpen their understanding of the key components and behaviour of a health-related issue as a complex system [6, 21, 22]. Once commissioned, these models allow decisions makers to draw on and learn from this joint understanding to better inform their policy and practice decisions [6, 9, 15, 21, 22, 28–30] and further model development and modification, post-commissioning, facilitates ongoing learning [6].

Participatory modelling approaches are an important feature of system dynamics modelling and have been widely adopted in environmental modelling projects [1, 26, 27, 30–39]. Many guidelines and principles for participatory modelling have been developed with varying degrees of prescriptive detail [30, 32, 36, 40, 41]. The guidelines commonly emphasise the principles of: careful planning for stakeholder engagement; awareness and management of social and group dynamics; flexibility and responsiveness to stakeholder input; iterating and refining, being open and transparent; accepting uncertainty; and encouraging learning through theory building and hypothesis testing [30, 32, 36–43]. The implementation of these principles of participatory modelling processes are often not well described, or only reported in narrowly defined discipline-specific forums (e.g. system dynamics projects reported in system dynamics journals), thus limiting opportunities for interdisciplinary learning for public health policy and practice [25, 44, 45].

Many participatory modelling projects have focussed efforts on qualitative mapping or semi-quantitative modelling of systems using methods including fuzzy cognitive mapping, rich picture diagrams, causal loop diagrams and systems structure diagrams [1, 23–26]. Understanding the process of transforming these representations into quantitative models is important, particularly for complex, quantitative models developed with an inter-disciplinary participant group, such as the one described in this case study [1, 36, 41]. More detailed understanding is needed about the participatory modelling process and the impact of facilitators and constraints [25]. Recent multi-method and systematic reviews of knowledge mobilisation and participatory dynamic simulation modelling across health and other sectors also conclude that more knowledge is needed about which approaches work best, in what settings, and how and why they are effective [42, 46, 47]. Effective learning about the future role of systems approaches will come from natural experiments and case-studies, and the field of knowledge mobilisation will benefit from empirical studies of participatory modelling in applied ‘real-world’ settings [46, 48, 49].

Three case studies, focusing on alcohol related harms, childhood overweight and diabetes in pregnancy, utilising participatory dynamic simulation modelling methods have been implemented in Australian health policy settings [50–55]. Key aspects and activities of the novel participatory modelling methods used to collaboratively develop qualitative representations of the complex systems being modelled; participant experiences of the modelling process; and the model outputs and their application as decision support tools have been described elsewhere [50, 51, 53, 56, 57]. This paper focuses on the diabetes in pregnancy case study. It reports the findings of a qualitative analysis undertaken to examine the stakeholder deliberations, analytic processes, and decisions involved in using a participatory process to transform qualitative conceptual maps of diabetes in pregnancy into a quantified dynamic simulation model.

### Case study context

Diabetes in pregnancy (DIP) is a complication of pregnancy that is defined as carbohydrate intolerance resulting in hyperglycaemia (abnormally high blood sugar). It includes women for whom the first recognition or onset of the condition occurs during pregnancy, as well as women with pre-existing type 1 and type 2 diabetes mellitus [58]. The prevalence of DIP is increasing both in Australia and internationally [59], and increasing the burden on the health care system. Approximately 16% of women who gave birth in the Australian Capital Territory (the case study focus region) in 2016 were diagnosed with diabetes in pregnancy, increasing from 6% in 2008 [60]. There are short- and long-term health risks for both mother and baby, including increased risk of birth injury in the short term and development of diabetes later in life [61–64]. The available evidence does not definitively guide health services on how best to prevent and manage DIP. For example, questions regarding the timing and methods of prevention and screening, criteria for diagnosis, targets for treatment and differential effects of treatment are all current challenges for DIP policy and treatment planning [65–68]. These issues cross the spectrum from specialised clinical management to population health interventions and such policy and service decisions are likely to benefit from sophisticated analytical tools, such as dynamic simulation modelling.

## Methods

The qualitative study involved analysis of data methodically collected during the participatory process for the development of a dynamic simulation model for diabetes in pregnancy (the case study). The case study (Box 1) and the participatory modelling process (Box 2) are described below to provide background contextual information. The data sources and qualitative analysis methods for this study are described below.

**Box 1: Case study description**

Researchers partnered with an Australian jurisdictional health department, and a multidisciplinary group of stakeholders including clinicians, health economists, public health practitioners, simulation modelling experts and health policy decision makers, to co-produce a sophisticated, multiscale dynamic simulation model to support health policy and practice decisions for diabetes in pregnancy. The case study, participants, key project roles and participatory processes have been described in detail elsewhere [51, 54, 57].

The hybrid model was developed between 2016 and 2018 and integrates multiple modelling methods (agent-based, system dynamics and discrete event simulation modelling—see the following references for more information about these modelling methods [32, 69–72]). The purpose of the model was to explore short- and long-term implications of rising rates of diabetes in pregnancy and associated risk factors. The model simulates alternative policy, program, and clinical intervention scenarios to inform prevention and management decisions [51, 54].

**Box 2: Participatory modelling process**

An overview of the approach used to build the dynamic simulation models using participatory methods has been described previously [51]. Broadly, this involves an iterative process of convening expert stakeholders, conceptual problem mapping, synthesising evidence, quantifying the key dynamic relationships within the system, presenting model versions to participants and end users, refining the model, and applying the model to support evidence-informed dialogues about policy options.

The end-user participants were central to the model development process. Contact was initiated early and engagement was negotiated to ensure that the scope of the model reflected key policy and planning questions, the interaction of key risk factors, and context specific intervention priorities [51]. The participatory process involved workshops, web-based and face-to-face meetings and ongoing communication via email or telephone. Participants had differing levels of intensity and duration of involvement in the project, ranging from those who contributed to group activities primarily as workshop participants, to others who also contributed as workshop facilitators, attended the regular project team meetings, and facilitated subsequent communications about the application of the model.

**Fig 1:**
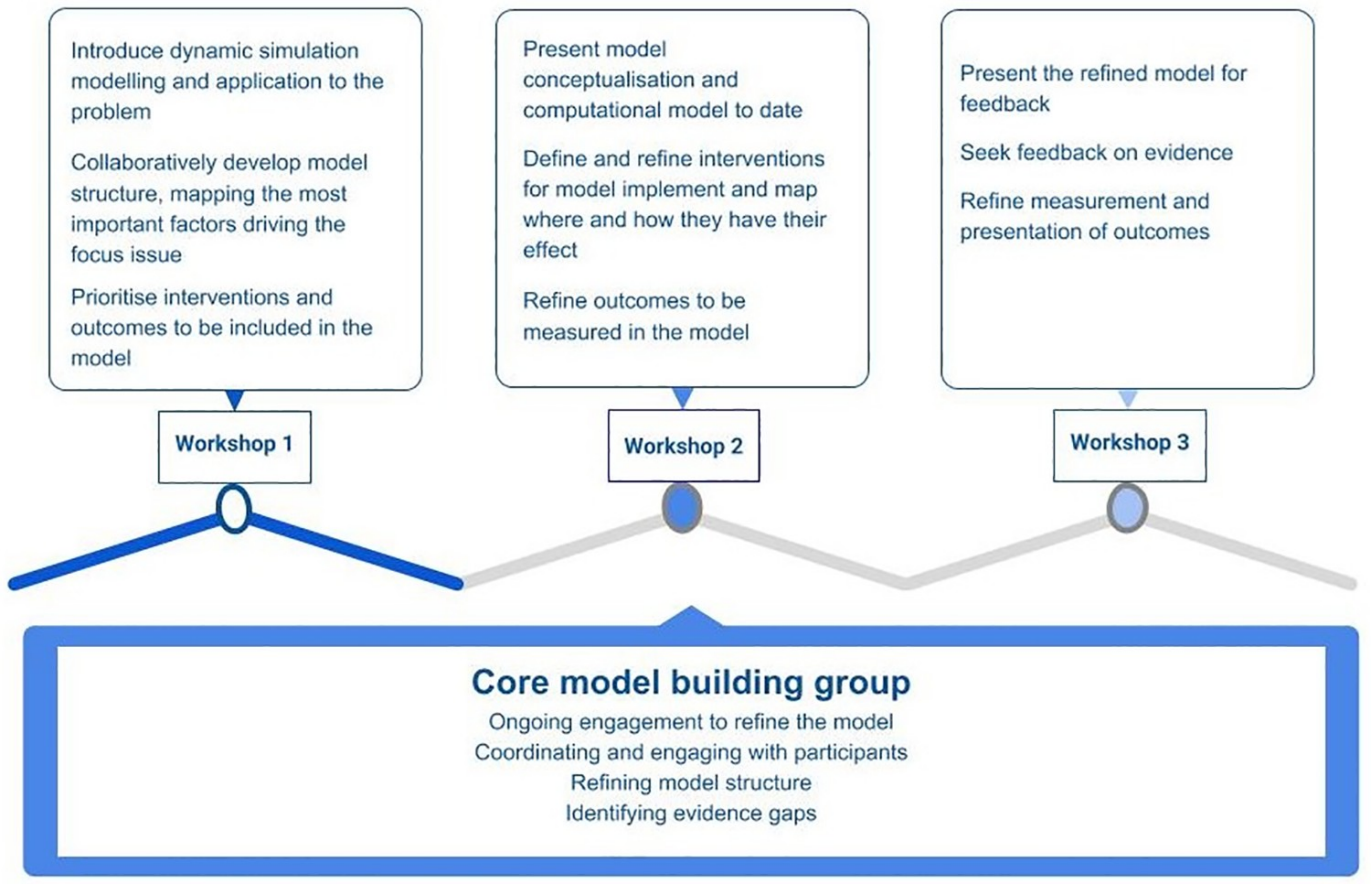
Overview of activities involved in the participatory process.

An overview of the activities involved in the participatory process is presented in Fig 1. Workshops were conducted where participants interacted and engaged in group activities to develop conceptual maps of the factors contributing to diabetes in pregnancy and its potential outcomes. During the workshops, they also discussed the quality and availability of evidence to inform the model development, prioritised interventions and outcomes to be explored in the model, and provided feedback to refine the model. The model development process was iterative at every stage, with the core model building team gathering information from participants, integrating it with other evidence and data sources to inform the model development process and receiving feedback from participants before proceeding to the next step (Fig 1). Interaction with participants also occurred between workshops, and continued for some months after the final workshop. In the later stages of model development, the iterative feedback process centred around the presentation and discussion of the model results.

### Data sources

Data sources for preparing this paper included recordings of participatory workshops (n = 3), web-based meetings with participants (n = 3) and model development meetings (n = 3) with the core modelling team. The face to face meetings were audio recorded and photographed and the web-based meetings were audio-visually recorded. The core modelling group comprised of 11 people including computer scientists, computer science students, public health practitioners and medical specialists. LF, JA, GM, NO and PK were members of the core modelling group. Key meetings with members of the core model development group were audio recorded and one was professionally transcribed as a resource to facilitate the documentation of the model. Additional data included the written records and field notes from model development meetings, including a key modelling team meeting held after the first workshop where the qualitative conceptual map was synthesised. The field notes were based on observations of the participatory workshops, workshop debriefing discussions with research officers (EO, NR, JD and CW, see Acknowledgements) and reflexive discussions regarding the model development process between the authors. Email communications with participants were also compiled for triangulation with the other data.

### Data coding and analysis

The analysis presented in this paper builds on previous work focusing on the experiences and perceptions of decision makers who engaged in the participatory modelling processes [57]. The previous analysis was conducted using grounded theory, whereas this data coding and analysis used thematic analysis focusing on the research questions outlined below. It was guided by the “theoretical” approach to thematic analysis described by Braun and Clarke [73] with the focus being guided by the researcher’s analytic interest, and therefore more explicitly researcher driven than inductive coding and analysis [73]. The thematic analysis focused on the problem solving and decision-making processes underlying the explicit activities in which stakeholders participated during the model development described in Fig 1. The analysis was guided by the following research questions: What were the key elements and features of the participatory approach that were required to successfully develop a policy relevant dynamic simulation model from a qualitative systems map? What types of questions were asked by the stakeholders, what concerns and issues were raised, and what was the feedback from participants during the process? What challenges and tensions arose in the process and how were they managed?

The audio-visual recordings were viewed, coded and analysed by the lead investigator (LF). Field notes, observations, records of reflexive discussions, email exchanges and recordings of meetings / workshops were analysed progressively by LF and discussed regularly with JA, and LR throughout the process. An iterative process of descriptive coding and analytical memos was used to develop themes and conceptual categories and explore their inter-relationships. Themes and insights were triangulated across the different data types and sources. The progressive analysis was further revised as new data was incorporated. Analytic memos written by LF were shared with JA and LR to facilitate the analysis review process. Vignettes based on data from the case study were written to demonstrate practical examples of important decision points and the processes used to develop model components.

### Ethics and consent to participate

This study was reviewed and approved as low risk by the ACT Health Human Research Ethics Committee (ACTHLR.15.150) and the University of Notre Dame Human Research Ethics Committee (0151195).

All participants gave individual written consent, were assured of confidentiality, and were free to withdraw from the study at any stage.

## Results

**Fig 2:**
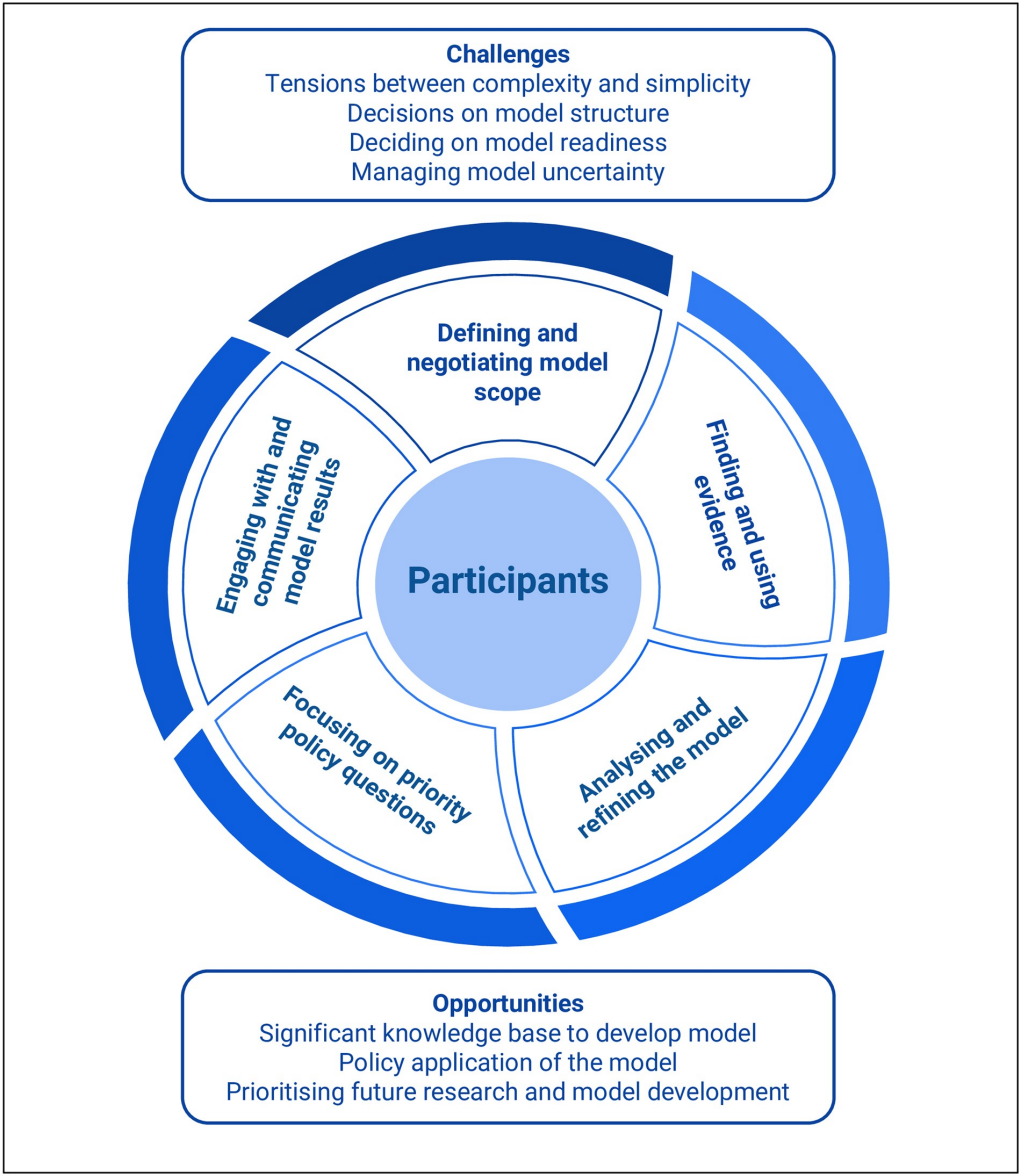
Overview of the analytical objectives and decision-making processes involved in the participatory development of a dynamic simulation model.

The qualitative analysis uncovered the iterative cycles of engagement, analysis, negotiation and refinement involved in the process of developing a dynamic simulation model as a quantified decision support tool for diabetes in pregnancy. The core analytical objectives and decision-making themes involved in the participatory model development process are described below and represented in Fig 2. In summary, the process of engaging with participants to develop a quantitative model involved five distinct phases including: (i) defining and negotiating the model scope; (ii) finding, critiquing and using evidence; (iii) analysing and refining the model; (iv) ensuring that the model remained focused on priority policy questions; and (v) engaging with, evaluating and communicating model outputs. Each of these phases are explained in detail below. The schematic diagram in Fig 2 illustrates how each of these conceptually and practically distinguishable aspects of model development involved interaction and engagement with participants at the centre of the process. However, it is important to note that these phases did not occur in any linear or chronological order. Instead interactions and discussions that occurred later in the model development process, as the model was analysed and refined, resulted in earlier phases being re-visited and refined or revised. The results section concludes with a description of the overarching challenges that arose from the participatory process itself, and the strategies used to overcome them, as well as the model application opportunities that resulted from the participatory process. A glossary explaining modelling terms is provided in the supplementary file: S1 Glossary.

### Defining model scope

**Fig 3:**
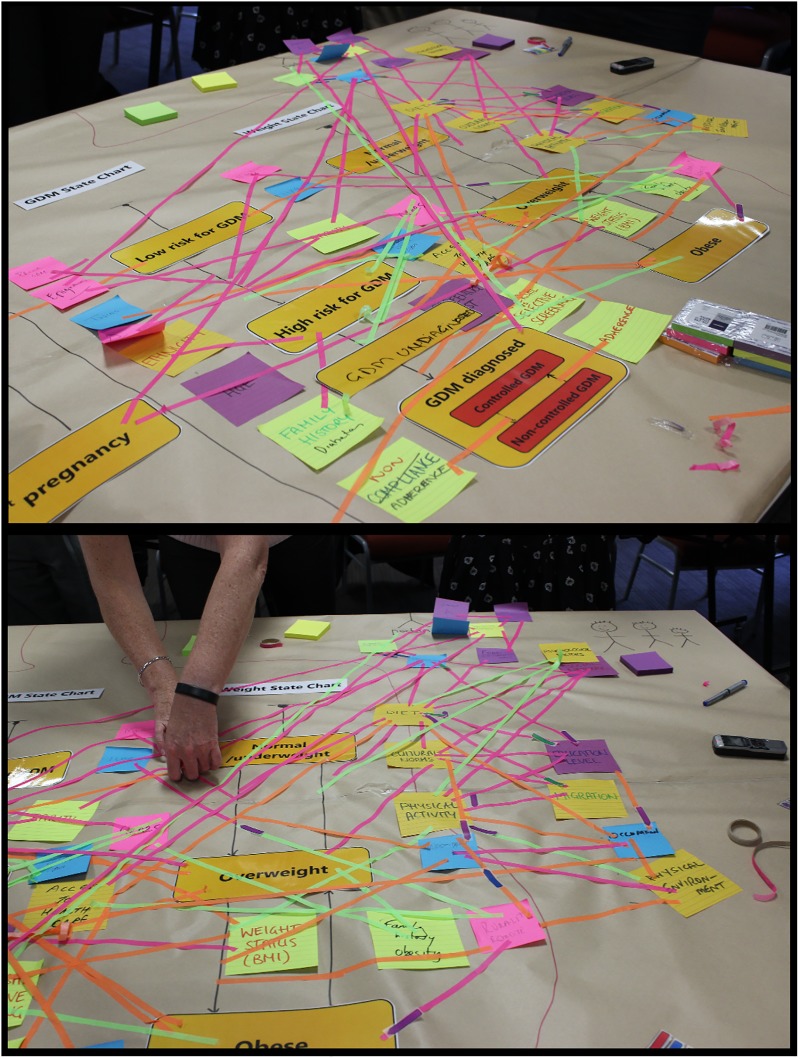
Problem conceptualisation map from participant workshop 1 (detail).

A primary aim of the first participant workshop was for the core model building team and workshop participants to jointly conceptualise and qualitatively map the ‘system’ of Diabetes in Pregnancy in the form of a ‘draft model structure’. In this instance, it was represented in the form of ‘state charts’ as used in agent-based modelling methods [51, 54]. State chart elements relating to diabetes in pregnancy were derived from discussions with participants prior to the first workshop and were pre-printed and presented as a draft model structure to facilitate the activity. Participants were invited to add to and modify the draft model structure and encouraged to highlight and explain the interconnections between the components of the system, any changes over time, feedback loops, and sources of inertia and delay. The problem conceptualisation for diabetes in pregnancy as it appeared at the end of Workshop 1 is shown in Fig 3.

The participants’ initial problem conceptualisation was a detailed, qualitative representation of the interacting factors contributing to the development of diabetes in pregnancy, jointly developed to incorporate the multiple perspectives of the expert participants. However, the initial map developed in workshop 1 (Fig 3) required further synthesis and refinement of its conceptual representation before it could be operationalised as a computational model. To achieve this, the core model development team, in subsequent consultation with the expert stakeholders, used the map and voice recordings of the mapping exercise to identify important themes, events and interconnections to be captured in the model. This involved systematically reviewing the diagram to determine the priority factors that influenced the postulated causal pathways, and the most important events and agents to be quantified in the model. These factors are presented in Table 1. For example, factors were prioritised for inclusion in the model if they were identified in multiple places in the concept map, or emphasised by stakeholders as influencing causal relationships between, and transitions within, the developed state charts.

**Table 1:** Factors influencing the development of diabetes in pregnancy prioritised from problem conceptualisation.

**Factor**	**Examples**
Family history / genetic factors	Family history of obesity or diabetes
Food environment / diet	Unhealthy diet, access to healthy foods, food security
Physical Activity	Level of physical activity or sedentary behaviour, physical environment
Health state	Diabetes in previous pregnancy, other obstetric risk factors, personal history high birthweight
Health care system	Universal or selective screening, access to health care, government policy
Metabolic functioning	Glycemic regulation, insulin sensitivity, weight status, gestational weight gain
Non-modifiable factors	Maternal age, high risk ethnicity, migration
Psychosocial factors	Social network, education level, cultural norms, psychological factors
**Events**	**Examples**
Medical interventions	Screening, specialist services, diabetogenic medications, bariatric surgery
**Model components**	**Examples**
Agent types	Mothers, babies, health care workers

The modelling methods used in the case study included system dynamics, agent-based and discrete event modelling—a decision that was primarily made by the technical expert modellers, in consultation with the others in core modelling group. The current understanding of the aetiology of diabetes in pregnancy (as described in Vignette 1 below) facilitated decision making about the modelling methods. Advances in computer simulation tools have meant that the multiple modelling methods mentioned above can be used in a single model, allowing focused selection of the most appropriate method to articulate different components of the model. This flexibility leveraged the advantages of each method without needing to constrain the representation with the limitations of a single method. Aggregate model components, such as with system dynamics, don’t allow for exploration into individual differences in predisposing factors, adherence to diet or medication, or other circumstances such as social determinants of an individual’s health. Therefore, agent-based modelling methods were chosen to enable the exploration of individual differences in predisposition and risk exposures. Agent-based modelling methods were also used to capture individual trajectories through risk exposures, inherited risk due to maternal history and ethnicity and consequent development of disease. A system dynamics stock and flow ageing chain structure was initially chosen to initialise and represent the population. Population members who met the definition for ‘high risk’, i.e. according to the Australian Diabetes in Pregnancy (ADIPS) definition, ‘budded’ from the aggregate stock and flow structure and became agents within the model. Agent-based modelling state charts were implemented to represent pregnancy transitions, weight transitions and the development of diabetes. Discrete events simulation components were implemented to represent agent use of health services.

### Analysing and refining the model to maximise relevance and utility

Versions of the model were presented back to participants at the second and third workshops and other web-based meetings to demonstrate how the core modelling group had operationalised the qualitative conceptual map of diabetes in pregnancy. The participants’ analysis and critique of the evolving model were an important contribution to improving the structure, and refining the causal pathways, and their underlying logic and assumptions.

**Fig 4:**
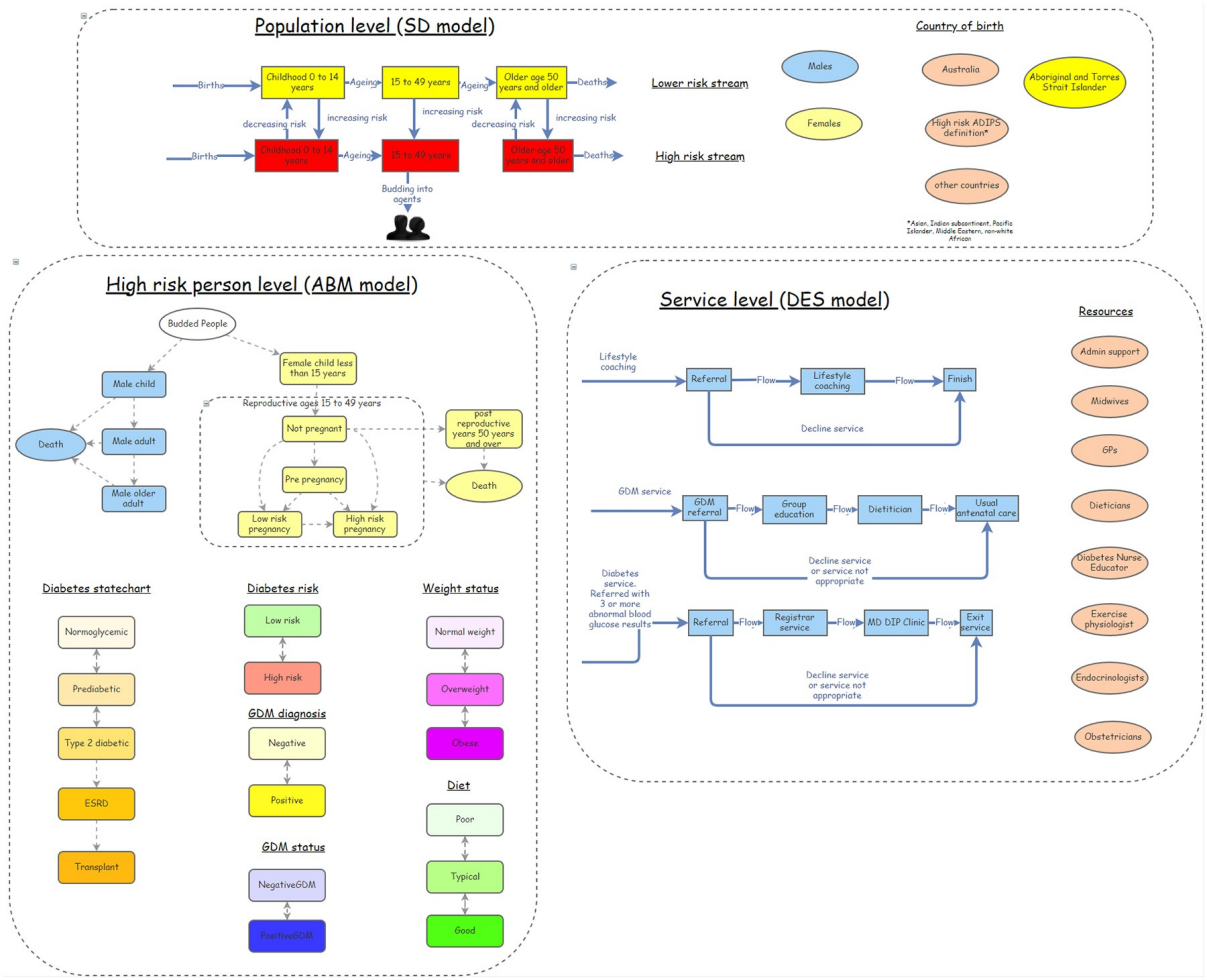
Simplified model structure presented in workshop 2.

We include here an illustrative example of how the evolving draft model was presented to participants in the second workshop using a simplified representation of the model elements in Insightmaker^™^ (Fig 4). The examples of agent”life stories”, presented as clinical case histories, were used to talk participants through the model structure and logic.

For the expert participants, particularly those from clinical backgrounds, the presentation of individual case histories, as “stories” from the model, was a familiar and well-understood method of communication. It provided an opportunity for participants to become familiar with a strategic view of the model, without becoming swamped by the detailed structures used in modelling software. Participants asked questions of the core modelling team, clarified the use of terminology, and helped to refine the model logic. They also provided feedback based on their clinical and policy expertise that identified important gaps in the model; for example, the need to incorporate a representation of the complex heterogeneity of diabetes aetiology as discussed in Vignette 1.

*Vignette 1*. *Improving the representation of the development of diabetes in pregnancy*
  *An issue raised frequently during workshops and meetings was the complex heterogeneity in the development of diabetes in pregnancy*. *Participants emphasised that the causal mechanisms for development of the condition were complex*, *multifaceted and an area requiring further knowledge development*. *For example*, *a baby may be born with diminished beta cell mass and function due to genetic predisposition*. *The intrauterine environment also impacts on risk; being exposed to dysglycemia (high blood sugars) in-utero can lead to short- and long-term effects on the baby including macrosomia*, *risk of high weight status in childhood and adult life and increased risk of early development of diabetes*. *The causal mechanism for diabetes development in some individuals was through increased insulin resistance*, *however for others*, *declining beta cell mass and function was the driving factor*. *A third group experience a combination of both*. *These causal mechanisms were also influenced by non-modifiable factors*, *such as ageing*, *and modifiable factors*, *including weight status*, *diet*, *and physical activity levels*.

**Fig 5:**
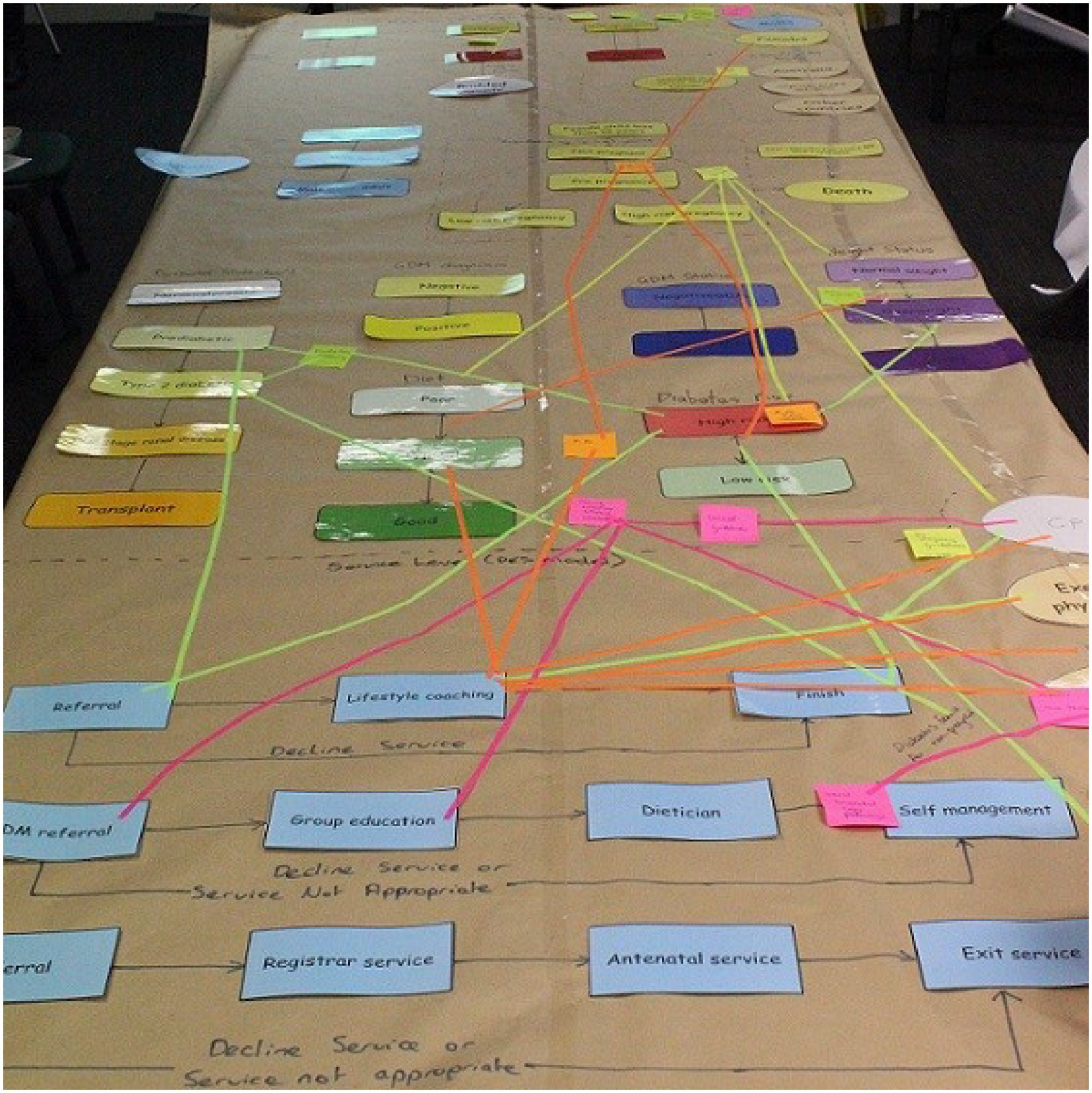
Intervention mapping to model architecture from workshop 2.

The definitions used in the model were aligned wherever possible with those used in accepted clinical guidelines, and the collaborative process of deciding on the terms and definitions helped to facilitate shared understanding of these within the group. Participants also proposed credible assumptions to be used in the model e.g. all women from ethnic groups defined by ADIPS as high risk should be defined as “high risk” in the model. Versions of the simplified model were printed and used in small group activities in workshop 2 to map directly to the model architecture the prioritised interventions, as identified by the group (Fig 5). This decision making process was aided by technological advancements in the user interface of the selected modelling software, so that ‘state charts’, ‘action charts’, ‘stocks and flows’, and process modelling components can be used to replace thousands of lines of code to clearly visualise and communicate model logic and thereby facilitated transparency and enabled stakeholders to meaningfully critique the model.

The repeated opportunities for stakeholder participants to actively interact with and discuss the model allowed them to test the evolving model structure against their “real-life” professional experience of working in diabetes in pregnancy research, policy and practice. It also allowed the modellers to test their own understanding of the issue (and how this knowledge had guided their technical model development) against the knowledge of content experts working in the field. The multi-disciplinary group of health sector participants brought to modelling discussions a breadth and depth of knowledge and rich experience regarding the issue that would be impossible to gain from reviewing the data / literature alone. Participants were able to contextualise the logic and structure of the model, identify additional questions and data to be investigated, and additional factors to consider for inclusion.

### Finding and using the best available evidence

Over the duration of the model development process many published studies and other evidence sources were synthesised and used to inform assumptions and parameter values in the model. Participants were motivated to understand and review the data and evidence utilised and demonstrated their strong commitment to this process by continuing to engage and respond to requests for evidence. The potential sources of data and evidence that could be used to inform the model were, therefore, an important focus for discussion at workshops, meetings and out of session communication. Participants drew on their extensive knowledge of the literature, identified and explained the most relevant studies and their main findings, and offered advice to the modelling team about local population characteristics, exposures, and service variations that contextualised published study results. Importantly, participants also identified limitations of the available evidence and data, such as quality concerns about identification of diabetes in health service administrative datasets.

Agent-based models are valuable to explore individual differences in disease aetiology; however, they can have substantial data needs, and complex models like the diabetes in pregnancy model require quantification of many parameters and relationships between model components. Requests for evidence were circulated to the participant group for discussion as they arose during the model development. There were many requests for evidence that were identified by the core modelling team and discussed with participants during the model development process. Some examples include:

1. What is the probability of adverse perinatal outcomes for women according to their level of glycemic control during pregnancy (normal through to high levels of dysglycemia)?
2. Relating to the mechanism by which exercise affects insulin sensitivity—is it direct or moderated through weight status? Can physical activity have a positive impact on metabolic function but no impact on weight status?
3. What is the effect of insulin during pregnancy and does it differ from pre- or post-pregnancy?

These questions were framed with a brief contextual explanation of the model pathways and structures that required the additional information. Where possible, participants answered these questions by referring the core modelling group to quality published studies, including randomised control trials and prospective longitudinal outcomes studies where available; providing health service administrative data; or providing expert advice based on their extensive experience. The core modelling group also independently searched the literature for evidence and conferred with the expert participants about the robustness and appropriateness of the evidence identified before and while it was used to inform model development.

The expert participants were also able to critique and identify limitations in the published literature and health service data as well as knowledge gaps. For example, the health service routinely collects perinatal statistics with respect to perinatal outcomes, such as birth weight and admission to neonatal intensive care, however, only diagnosis of DIP is recorded and not level of glycemic control during pregnancy. These data were therefore unable to directly inform relationships between glycemic control and perinatal outcomes to answer question 1 above; and more detailed studies in the published literature were utilised instead. Where the published evidence was relevant but not specific enough to apply to the local context, it was often used to assist with calibration or validation rather than used as input parameters i.e. it was used to evaluate the model behaviour rather than as evidence incorporated into the model equations.

A common question that arose during the model development process was what to do when there was insufficient local data or other published evidence to inform the model structure or parameterisation. Strategies such as calibration of key parameters using historic trends for diabetes in pregnancy incidence and sensitivity analysis were utilised. These strategies are established in modelling literature and practice as robust methods to address these common modelling challenges but were unfamiliar to many participants. However, the mutual respect that had developed between participants and the core modelling team and the recognised value of dynamic modelling as a learning tool were helpful sources of confidence. The overall framing of the process was that dynamic simulation modelling is a tool that allows contributors to articulate a hypothesis of complex causal pathways in the emergence and progression of disease (including possible latent factors) by bringing together best available evidence and data, and then testing and refining that hypothesis through computation, simulation and validation against real-world historic data patterns.

Sensitivity analysis was used to determine which uncertain parameter estimates were most important to the outcomes of interest, which informed priorities for future research. This identification of future research priorities was another function of the modelling process that was highly valued by the participants.

Finally, the DIP model also utilised, as sources of evidence, the existing diabetes modelling literature. For example, existing, peer-reviewed mathematical models of diabetes progression were presented and explained to the clinical and policy expert participants for consideration as evidence to help quantify parameter estimates and equations, such as those representing variations in the development of diabetes. These mathematical models enabled the modellers to quantify and operationalise the latent variables and causal mechanisms underlying the heterogenous development of diabetes, an identified gap in clinical diabetes research. Grounding the model in this established, and peer-reviewed, mathematical literature also enhanced the rigour and reliability of the model outputs.

### Focusing the model on priority policy and program questions

The model was primarily developed as a planning tool for exploring the resource implications and service costs of alternative policy and program options. Participant feedback guided decisions about which components of the larger system model of DIP would be prioritised for these health service decisions. They selected alternative health service options and service pathways as a priority for inclusion in the model.

The expertise of the participant group grounded the model in the real-world experience of intervention effectiveness. For example, studies of interventions delivered during pregnancy to prevent the development of diabetes have yielded disappointing results, and it was deemed important for the model to be able to compare early intervention options. Pre-pregnancy and inter-pregnancy interventions were prioritised for inclusion in the model; both at the population level, and those targeting high risk women. The mechanisms for impact were mapped to the printed model structure during the workshops and subsequent discussions. Participants indicated the transitions, states, parameters and other structures that were likely to be impacted by each intervention. For example, for interventions targeting weight loss, the impact on the weight status state chart were discussed by the group, and then mapped to indicate how this could flow through to impact on other model structures. These discussions with participants guided the core modelling group where to focus their efforts to ensure that the necessary components were operationalised to allow the most important policy and program questions to be explored (Vignette 2). Based on the detailed understanding of the expert participants, the structure of the model captured the impact of duration and level of exposure to dysglycemia on beta cell function for individual agents, and thus enabled the testing of both clinical and lifestyle intervention strategies targeted at different stages of the life course.

*Vignette 2*. *Accurately capturing impact of prolonged exposure to dysglycemia on intervention effectiveness*
  *Participant input emphasised that the model needed to account for the length of exposure to dysglycemia as this has a significant impact on intervention effectiveness*. *When a person is exposed to dysglycemia for an extended period of time*, *they lose effective beta cell function*, *and therefore*, *the ability to recover glycemic control even after engaging in an intervention*. *In contrast*, *an individual who has just been newly diagnosed with impaired glucose regulation or diabetes in pregnancy can recover glycemic control if they engage in physical activity or dietary modifications that lower their blood sugar levels and reduce damage to their beta cell function*. *Early interventions*, *for example*, *for a woman who experiences gestational diabetes in her first pregnancy*, *may therefore have more effectiveness than interventions for people with prolonged exposure to poor glycemic regulation*.
  *These important policy and planning questions focused on the underlying physiological mechanisms impacting on intervention effectiveness*. *They motivated the development of more detailed model mechanisms to capture the impact of actualised glycemic control on both maternal and perinatal outcomes*. *A detailed articulation of glycemic control*, *rather than simply considering the diagnostic status of an individual agent in broad terms*, *was required for the model to robustly explore clinical intervention scenarios of interest to participants*.

### Engaging with and communicating results and applying the model

Being open to and welcoming critique from diabetes experts was key to genuine co-design of the model. It was also important to ‘socialise’ the results of the model; that is, to test them against the knowledge and experience of the participant group. Viewing and discussing model results / outputs was a critical phase of the model development process and was essential to more fully elicit the expert knowledge of participants. Two types of knowledge were elicited through discussion of model outputs, namely: tacit expert knowledge, i.e. the knowledge that people generally won’t mention unless prompted; and explicitly considered expert knowledge that couldn’t be applied to the model directly, i.e. knowledge that wasn’t reducible to any one parameter or assumption and instead reflected the emergent behaviour of the system. In both cases, the elicited knowledge served as key sources of evidence to challenge the working dynamic hypothesis captured in the model. Simulation experiments enabled examination of the logical implications of the hypothesis, represented in the model structure, logic and assumptions, by exposing the performance of the model for outcomes of interest. For example, increases or decreases in insulin sensitivity occurred for individual agents in association with other physiological changes, such as pregnancy or weight gain or loss. This was consistent with the elicited knowledge from participants and the empirical evidence.

Viewing and discussing the model results also ensured that the model had fidelity i.e. that it produced results that were consistent with retrospective data and considered plausible by experts working in the field. These discussions emphasised that the model was not a “crystal ball” that would discern the future with pinpoint accuracy but could be used to make robust forecasts and enhance understanding about the relative value of alternative policy and planning choices. Full transparency about how the model scope was defined, and the limitations of the underlying data, also ensured that the participants were informed about its strengths and limitations, and thus more confident to make decisions about its application and value.

Storytelling was an important communication tool used in the model building process, and in discussing model outputs. The “life stories” of agents in the model were used throughout the participatory process to communicate the model structure and its capacity to demonstrate health outcomes at an individual level. Agents in the model were born with a risk profile based on both their mother’s history and her glycemic control during pregnancy. The agents aged during the model run time (80 years), gained and/or lost weight, underwent lifestyle and medical interventions, and experienced their own pregnancies. The model captured information (outputs) for individual agent health outcomes that both influenced feedback loops within the model and could also be used to report statistics from the model. This functionality offered great power to support telling rich and compelling stories that illustrated the textured evolution of agents over time. The presentations of individual trajectories were an effective communication tool to improve participant understanding of the model structure and logic. The communication of agent stories as “case histories” facilitated the ability of participants to relate the model logic and assumptions to their real-world experience providing services to women with diabetes in pregnancy. The process prompted questions and comments and facilitated participants’ engagement in analysing, refining and informing the model.

Storytelling for individual agents was also viewed by the expert participants as a valuable tool to communicate model results to a broader, less technical, audience. During discussions about the model outputs, participants identified that presentation of the knowledge gained from the model development process, as well as the results it produced, would be a critical determinant of knowledge mobilisation and communication with a broader audience. But they also reported that despite the improved transparency of the new software interfaces, the sophisticated and highly technical nature of the model would be a barrier to developing clear and easy to understand policy messages. Thus, supplementing model outputs with storytelling about individual patient journeys was viewed as a powerful tool to ensure that the results were relatable and easily understood. A plain language fact sheet was developed for the model incorporating both real-world and individual agent stories and is available at: https://preventioncentre.org.au/wp-content/uploads/2018/08/080818_Diabetes_FactSheet.pdf and a podcast was also made to communicate the project to a broader audience, available here: https://preventioncentre.org.au/resources/tackling-the-pandemic-of-diabetes-in-pregnancy/.

### Feedback and iteration

An important overarching theme derived from these findings was that of continual feedback and iteration, in which decisions about model logic and structure were regularly re-visited as new information became available. This is also represented in the configuration of Fig 2 in which the processes of model development fit together as non-linear phases. For example, as noted above, the process of participants viewing and discussing individual agent stories, and engaging with results from the model, elicited additional information and developed new forms of shared knowledge. This additional information and knowledge were then considered for incorporation into the representation of causal pathways and other model components. This led to further refinement of the model, and identified the need for additional evidence to inform that refinement. The highly iterative nature of the participatory process resulted in both challenges and opportunities that are discussed below.

### Overcoming the challenges that arise from the participatory process

1 **Tensions between model complexity and model simplicity**
  Desire for complexity and detailed representation—The expert participants had highly evolved and detailed knowledge about many aspects of diabetes in pregnancy; including disease aetiology, the technicalities of treatment and testing regimens, and complex health service delivery. It was common for the conversations to go deeply into complex details, for example, about service pathways, issues with diagnostic testing methods, and participation rates for screening. However, while such topics are important for real-world service delivery, they were often too detailed to be captured in the model. Thus, an important challenge for the participatory model development process was to distinguish which aspects of DIP were important to represent in detail, and which aspects could be left out or represented in a more stylised, or simplified, way. It was important to address the opportunity cost of including details and for the participants to prioritise only those aspects that were essential for more detailed inclusion. These discussions considered the extent to which the details would be needed to adequately represent intervention mechanisms, and their outcomes, and the likely pathways of impact for the prioritised policy and practice questions. A road map analogy was an effective communication tool to facilitate these discussions, i.e. like a road map, the model needed to include essential landmarks to make it fit-for-purpose and did not need to include every tree or driveway along the route. When particular details were considered important by some individuals but could not be prioritised in the agreed scope of the model, they were recorded as opportunities for future model expansion in subsequent projects.
  Desire for speed and simplicity—a contrasting challenge was the tension between developing a sophisticated and highly articulated model that could reliably and plausibly evaluate the interventions of interest to participants and their co-existing desire for a simpler, faster model both in terms of development and running time. In these circumstances, the onus was on the core modelling group to balance this tension between complexity and simplicity and determine the “minimal viable model”. The minimum viable model is the simplest solution that has the requisite robustness, completeness and reliability to rigorously address the participant needs. These negotiations and decisions relied on the extensive knowledge and experience of the lead modeller to ensure that the model developed was robust and rigorous considering these pressures.
2 **Ensuring the model design and structure are appropriate**
  Decisions about how to represent prioritised factors in the model were challenging. An early version of the model incorporated a simplified, statistical representation of the interaction between risk factors. For example, an individual’s probability of developing diabetes in pregnancy was programmed as increasing according to a linear correlation with their count of risk factors. However, this representation was not dynamic, did not allow for other important elements, such as the length of exposure to dysglycemia, lacked the ability to robustly capture the effects of counter-factual interventions, and limited the use of the model to explore the combination and interaction of intervention options in the development of DIP. Later versions of the model used endogenous or latent variables to represent the causal physiological mechanisms, thus allowing exploration of complex interactions between risk factors, and the exploration of counterfactuals.

The use of endogenous or latent variables created challenges in the interpretation of model outputs. For example, it was challenging on occasions when the model outputs didn’t produce familiar or expected results, e.g. when the emergent outcomes were counterintuitive. This was managed by identifying model outputs that could readily be checked against historic trends and empirical evidence, which reassured the participants when the model reliably replicated existing data. Unexpected results from the model also provided an opportunity to explore the logic and assumptions of the model and make improvements. For example, model results showed DIP incidence plateauing in contrast to the increasing rates observed in administrative data, and this led to an investigation of possible explanations. The investigation explored whether the plateau effect was due to the length of the ‘burn-in’ period used in the model and different burn-in lengths were tested to assess their impact. The impact of the representation of weight dynamics was also examined, leading to further changes as detailed in Vignette 3 below. Participant discussions regarding unexpected results also helped to identify quality issues and anomalies affecting the administrative data used to determine historic trends. For example, variations in the implementation of changes to the blood glucose standard used for diagnosing diabetes in pregnancy and changes to diagnostic testing assays impacted historic incidence rates leading to rapid increases. These artefactual increases resulted from process changes rather than changes to the underlying population rate of diabetes in pregnancy and it was important to consider this when assessing the model results against trends in administrative data.

*Vignette 3*: *Challenges in representing weight dynamics*
  *High weight status is an important and modifiable risk factor for the development of diabetes in pregnancy*. *Weight status was identified in the initial problem conceptualisation and included in model versions from the inception*. *The representation of weight status evolved significantly through the participatory model development process*. *Initially weight status was represented as BMI categories in a state chart specifically characterising an individual as present in one of healthy weight*, *overweight and obese states*. *Each agent was assigned an initial state based on an age and ethnicity specific distribution and transitions between states occurred according to hazard rates*. *As the model evolved and interventions were prioritised*, *defined and quantified*, *it became evident that a more detailed representation of weight status would be required*.
  *The representation needed to capture*:
  - *Intervention effects that resulted in weight loss for an agent but were insufficient to move that agent from one BMI category to another i*.*e*. *a weight loss of five kilograms may reduce an agents BMI by one or two units but may not move them from an obese to an overweight state*.
  - Dynamics in weight status across the life course
  - Population changes in weight distributions over time
  - *Impact of weight status on physiology underlying the development of diabetes in pregnancy*, *particularly on insulin resistance*, *and distinct effects during and outside of pregnancy*.
*The representation of weight status was evolved to capture agent weight as a continuous variable that changed dynamically with age and pregnancy events based on published evidence*. *An agent’s weight status (BMI) impacts on their insulin sensitivity with increasing weight leading to decreasing insulin sensitivity*.

3 **Deciding when the model is ready**
  Dynamic simulation models can always be further refined and improved. Another important challenge arising from the participatory process was achieving consensus on when the results were “good enough” to inform decision making. This decision was primarily informed by the following considerations:
  1. Reliability—How reliably the model results matched historic data trends across a range of indicators, including diabetes in pregnancy incidence overall and for important subgroups; population weight status categories over time; and general demographics such as age structure.
  2. Completeness—How satisfied the core modelling team were that they had captured the most salient aspects of the issue in enough detail to robustly explore policy questions.
  3. Experimentation—did the model produce plausible results during scenario testing of interventions, i.e. did the simulated intervention scenarios producing results that had face validity among participants who had extensive professional expertise in diabetes in pregnancy and sound knowledge of relevant research?
  4. Timing—having the model results ready in time to be used in policy dialogues.
  5. Acceptability—Was there sufficient acceptance of the fidelity and plausibility of results produced by the model among the expert participants? Were significant concerns raised and adequately addressed?
4 **Being transparent about uncertainty**
  It was also important to be transparent with participants about model uncertainty, for example, differentiating parameters based on quality, comprehensive evidence and those where the evidence was less certain. Sensitivity analyses determined how influential the parameters were on the model results. This information was shared with participants and discussions focused on either identifying new studies that could be utilised or confirming that the evidence gaps still existed and were therefore a priority for future research.

### Opportunities arising from the participatory process

The participants in this case study were nationally and internationally acknowledged experts and included health professionals who were embedded in local service provision and policy decision making. The participatory model development process included drawing on the participants’ networks to socialise the model to other decision makers, who had not been involved in the process. The participants also identified opportunities for the model to be presented and applied as a decision support tool for policy and programs. Through their professional networks, the participant group facilitated new relationships and useful leads for additional expertise and evidence to improve the model. Participants continued their engagement with the model after the formal activities of the process were finalised and advocated for the model to be used in policy decision making.

In summary, the participatory process resulted in a robust, highly transparent model with an agile, responsive design. The multiple modes of engagement and interaction with participants provided a built-in peer review-like process to ensure that the model was valid and fit for purpose. The network of participants involved in the project also facilitated the identification of new priorities and opportunities for research and further model development.

## Discussion

The primary goal of participatory dynamic simulation modelling is to provide decision support and facilitation in planning and policy contexts. The initial exploration of diabetes in pregnancy conducted at the commencement of the model development process resulted in a qualitative conceptual map that was complex, not yet well-defined, and of limited value for guiding policy. Through a deliberative participatory process that included synthesis and exchange of data and information, and iterative cycles of negotiation and refinement, a quantified decision support tool was developed. To fully understand and evaluate the rationale and logic of an participatory modelling process, both the interaction among the model building group, and the relationship between the participatory process and the decision context needs to be described [25]. The key elements of an interdisciplinary, participatory approach to develop a dynamic simulation model for diabetes in pregnancy included: determining the focus topic; defining the model scope; iteratively refining the model structure and logic; reviewing and using evidence; ensuring that the model was focused on priority policy questions; communicating results; and applying the model to inform health policy decision. The decisions required were highly interactive; with participants engaged via multiple forums e.g. workshops, web meetings, emails, and small group meetings. Participants identified important sources of evidence to inform model parameters and assumptions. The professional networks available through the participant groups ensured that the model was focused on current, priority policy questions and initiated opportunities for it to be applied in practice. Storytelling was an effective strategy for facilitating participant understanding of the structure and logic of this complex model and to communicate model results to a wider policy audience.

A new framework for reporting participatory modelling projects has been proposed within the environmental modelling field as a tool to facilitate sharing of knowledge about the participatory process and stimulate innovation [25]. The 4Ps framework has highlighted the need to describe “how” participants are involved in model development: firstly, to contribute to the interpretation of models developed using participatory methods; and secondly, to facilitate learning about participatory modelling tools and strategies [1, 25]. The 4Ps framework identifies purpose, process, partnerships and products as key dimensions of participatory modelling projects and practices: (1) the Purpose for selecting a PM approach (the why); (2) the Process by which the participants were involved in model building (the how); (3) the Partnerships that formed around different parts of the process (the who); and (4) the Products resulting from these efforts (the what) [25]. Our analysis from the DIP case study falls within the Process component of the 4 Ps framework in that it explored how the participants were involved in the model building process. Three questions are raised in this component: What were the characteristics of the interaction between the participants and the model? What was the level of participation? What was the relationship between the participatory modelling and decision-making processes? [25]. We consider these questions below in relation to the DIP case study and other modelling literature.

### Contribution of expertise to develop and refine the model

Participant interactions contributed significant expertise and local context knowledge to the development and refinement of the model. Advances in modelling software are improving the visual representation of model components, making them easier to use, and more transparent to stakeholders not trained in modelling [6, 42]. This facilitates a participatory process by which the significant combined knowledge of expert groups can be applied to model development [6]. By repeatedly exposing and explaining the underlying model components in workshops and meetings, the participants in this case study were able to understand, analyse and refine the overall logic and structure of the model. They were able to identify areas where more detail was required or where assumptions could be improved. However, their involvement in decision making about the type of modelling methods used was limited e.g. which factors to represent using system dynamics vs agent-based modelling components. As health stakeholders become more experienced with dynamic simulation modelling, the potential will increase for them to contribute to technical decision-making regarding modelling methods. The experience and knowledge developed by participants’ in this case study may enable them to even more confidently and effectively contribute to future modelling projects.

Incorporating participatory processes in simulation modelling also facilitates learning by building shared a understanding of the problem and potential solutions, and which is refined with data and evidence through group interactions [36, 41, 74]. Through the exchange of information, knowledge is shared, and new knowledge is created, leading to changes in understanding [25, 36]. The interdisciplinary dialogue facilitates the sharing of different types of knowledge on critical issues from a range of perspectives [36, 44, 52].

The model developed in this case study utilised and integrated diverse evidence sources to quantitatively operationalise a theory of the causal mechanisms of intergenerational, social, cultural, economic and environmental factors that influenced behaviour and development of diabetes in pregnancy based on the qualitative map developed interactively with participants. Model assumptions and parameter values were derived through a process of evaluating and critiquing the many sources of evidence, including those considered both at the top e.g. systematic reviews and meta-analyses, and the bottom, e.g. case reports, of traditional evidence hierarchies [52, 75]. Integration and triangulation of evidence from systematic reviews, local analytic studies, conceptual models, and expert and local knowledge was required to map and quantify a broad range of complex public health issues [19, 52]. The model simulations allowed robust examination of the logical and quantified consequences of the postulated dynamic causal hypotheses and to test the impact of policy and planning decisions and counterfactuals using experimentation.

### Participants were highly engaged in the co-production process

Stakeholder input and acceptance are important factors in increasing the usefulness and application of models [25, 36, 42]. The degree of success of a participatory process can be discerned from stakeholders’ trust in modelers’ expertise and the amount and quality of information they give, as well as whether they intend to use the model and will participate in future collaborations [36]. Most participants in the case study reported here remained highly engaged throughout the project. They continued to contribute to discussions, attended meetings and were responsive to email communications. The level of interest in the model and associated communication products was high. Participants contributed advice on how to ensure the model could be applied to high priority policy questions and identified opportunities to facilitate its use in this context.

Models cannot comprehensively reflect the real world as details need to be omitted and boundaries defined around what is to be modelled [42, 71]. Highly-detailed models often require more data than is available; take longer to develop; can be difficult to calibrate and validate; and most importantly, they can be hard to understand [1, 42]. Both stakeholders and modellers can struggle with determining the level of detail to include and get drawn into trying to model reality instead of the decision essentials [42]. This challenge was evident throughout this case study. The model scope and level of abstraction was frequently re-visited and needed careful negotiation throughout the participatory process.

### Participatory modelling facilitated the use of the model for decision making

Finding effective strategies to communicate about both the model and the model results were an important challenge in this project. Modelling to inform policy relies on clearly explaining results, and their limitations, building confidence in the modelling process and outputs, and ensuring that the outputs are appropriately used [42, 57]. Active collaboration builds confidence in the model and enlists local champions for its application [42, 57]. The participatory process facilitated the identification of opportunities for making the model accessible to policy audiences, and strategies to address likely communication challenges. Opportunities to use the model to identify the policy options that were likely to have the greatest impact in local service planning were proposed. Participants were also interested in using the model to test whether highly advocated, but contested, interventions would be effective and or scalable to the population level.

Additional opportunities and potential applications of the model beyond the primary purpose of policy analysis were identified through the participatory interactions. For example, participants proposed that the model could be used to inform health education messaging by primary practitioners, such as demonstrating the risk of developing diabetes based on weight status, and the positive impact of engaging in lifestyle modification. This messaging was viewed as potentially leveraging women’s motivations to protect the health of their baby to encourage them to reduce their own risk profile pre-pregnancy and maintain good glucose control during pregnancy.

## Conclusion

The model developed in this case study moved beyond qualitative system mapping to a sophisticated, rigorously quantified, multi-method dynamic simulation model which represents the complex interrelationships underlying the development of diabetes in pregnancy. The challenges of the participatory process were outweighed by the benefits. The process allowed for the contribution of participants’ extensive and rich understanding of the issues, which was combined with the expertise of the modelling team to inform, analyse and refine the model logic and structure. The core analytical objectives and decision-making themes reported in this paper provide valuable insights for understanding and elucidating the process components of the 4Ps framework. Our analysis makes explicit the deep analytical work that occurs within the workshops, interactions and meetings of the participatory process. Like the workings underlying a clock face, the underpinning analytic processes are fundamental to participatory model development, but not readily observed without ‘lifting the lid’ through systematic data collection and analysis. In detailing the core analytical objectives and negotiations underpinning the participatory process, our findings provide unique insights for the planning and reporting of future participatory modelling projects.

## Supporting information

S1 Glossary (DOCX)
