# Supplementary material for: Turning conceptual systems maps into dynamic simulation models: An Australian case study for diabetes in pregnancy
Source: PLoS One. 2019 Jun 27;14(6):e0218875. doi: 10.1371/journal.pone.0218875 (PMC6597234; doi:10.1371/journal.pone.0218875)
Supplement: S1 Glossary — (DOCX) [file pone.0218875.s001.docx]

# **S1 Glossary**

| Term | Definition |  |
| --- | --- | --- |
| Ageing chain | A stock and flow structure used in system dynamics to represent the ageing of the population. Stocks represent a population in distinct age cohorts. |  |
| Agent | Agents in agent-based modelling may represent an individual object. Agents can represent almost any individual object, for example, people, vehicles, projects, products or countries [1]. |  |
| Agent-based modelling | A computer modelling method that simulates the actions and interactions of agents (i.e. individuals or collective entities such as organisations or groups) with a view to assessing their effects on the system as a whole [2].  This method is good at capturing heterogeneity in risk and in impacts of interventions and is able to capture social network influences. |  |
| Agent life story | This term was used to refer to the changes and events that occur to an agent throughout the simulation. For example, an agent will transition between states and in this model will experience increases and decreases in weight status, insulin sensitivity, glycemia and diabetes status. These changes are tracked within the model and can be analysed. |  |
| Budding | Budding is a technique used in hybrid modelling where agents of particular interest are “budded” or created from the system dynamics components and become individuals in the agent-based modelling components (for more information see <https://www.youtube.com/watch?v=8MKeHuV6cWs>). |  |
| Burn-in period | Burn in is a colloquial term that describes the practice of running the simulation model for a period of time to allow the behaviour of the model to stabilise. Results from this simulation period are not used. |  |
| Term | Definition | |
| Calibration | A process for tuning some parameters of the model so that the model’s behaviour in particular conditions matches a known (historical) pattern (https://help.anylogic.com/index.jsp). |  |
| Discrete event modelling | A modelling method that analyses processes and optimisation of resource allocation for service delivery (e.g. patient flows through an emergency department) [1]. | |
| Flows | Flows are also components used in system dynamics modelling. They are the rates at which the stocks (or system states) change. Flows are typically measurements of quantities in a given time period such as clients per month, dollars per year or incidence of disease during a defined period [2]. | |
| Hybrid model | A hybrid model uses multiple modelling methods within a single model [1]. For example, the model developed in this case study utilised system dynamics, agent based and discrete event modelling methods within one model. | |
| Initialisation | The set of parameter values used at the start of the simulation. | |
| Model structure | The manner in which the elements of a system are represented in the model or interrelated; the building blocks of the model including state charts, stock and flow diagrams and process diagrams. | |
| Multi-method model | See Hybrid model. | |
| Parameter | Parameters are used for quantifying characteristics of the modelled objects and relationships between them. A parameter is normally a constant in a single simulation and is changed only when the model behaviour needs to be adjusted (https://help.anylogic.com/index.jsp). | |
| Parameterisation | The implementation of parameters to quantify the model structure. | |

| Term | Definition |
| --- | --- |
| Robust | The term robust was used in this paper to refer to both the model and the evidence used to inform it. In this context robust was defined as being rigorous, reliable, replicable and defensible. |
| Sensitivity analysis | Sensitivity analysis is used to explore how sensitive the simulation results are to changes in the values of model parameters. The analysis runs the model multiple times varying one of the parameters and shows how the simulation output is impacted by the variation (https://help.anylogic.com/index.jsp). |
| State | Represents the “state” of the agent e.g. the agent is either in a pregnant state or not pregnant state. States are mutually exclusive and agents transition between states according to the state chart rules [1]. |
| State chart | A visual construct that allows the modeller to define the behaviour of agents using rules [1]. |
| Stocks | Stocks are components used in system dynamics modelling. They are accumulations and characterise the system state. Stocks are usually expressed in quantities such as people, inventory levels, money, or knowledge [2]. |
| System dynamics | System dynamics is a method for understanding how systems change. It models the relationships between elements in a system and how these relationships influence the behaviour of the system over time [1, 3-5]. Important elements of system dynamic models include feedback loops (the circular causality in the system), stocks and flows. |
| Transition | Transitions determine agent movements between states in a state chart. Transitions have triggers, such as a message, a condition, or a timeout that determine the agent state will change [1]. |

1. Borshchev A: **The Big Book of Simulation Modeling: Multimethod Modeling with AnyLogic 6**. Chicago: AnyLogic North America; 2013.

2. Grigoryev I: **AnyLogic 7 in three days**, 1st edn: AnyLogic; 2015.

3. Sterman JD: **Learning from evidence in a complex world**. *Am J Public Health* 2006, **96**(3):505-514.

4. Burke JG, Hassmiller Lich K, Neal JW, Meissner HI: **Enhancing dissemination and implementation research using systems science methods**. *International Journal of Behavioral Medicine* 2015, **22**(3):283-291.

5. Luke DA, Stamatakis KA: **Systems science methods in public health: dynamics, networks, and agents**. *Annu Rev Public Health* 2012, **33**:357-376.
